# The Ten Most Common Questions on Cytomegalovirus Infection in Hematopoietic Stem Cell Transplant Patients

**DOI:** 10.1007/s44228-022-00025-3

**Published:** 2022-12-29

**Authors:** Johnny Zakhour, Fatima Allaw, Sara F. Haddad, Souha S. Kanj

**Affiliations:** Internal Medicine Department, Infectious Diseases Division, Center of Infectious Disease Research, American University of Beirut Medical Center, Riad El Solh, PO Box 11-0236, Beirut, 1107 2020 Lebanon

**Keywords:** Hematopoietic stem cell transplantation, Cytomegalovirus, Preemptive therapy, Antimicrobial resistance

## Abstract

With the rising number of patients undergoing hematopoietic stem cell transplantation (HSCT), clinicians are more likely to encounter infectious complications in immunocompromised hosts, particularly cytomegalovirus (CMV) infection. Besides the high mortality of CMV end-organ disease, patients with detectable CMV viremia may have worse outcomes and decreased survival even in the absence of end-organ disease. In view of the implications on morbidity and mortality, clinicians should maintain a high index of suspicion and initiate antiviral drugs promptly when CMV infection is confirmed. High-risk patients should be identified in order to provide optimal management. Additionally, novel antiviral agents with a good safety profile and minor adverse events are now available for prophylaxis in high-risk patients and for treatment of resistant or refractory CMV infection. The following review provides concise, yet comprehensive, guidance on the burden and risk factors of CMV in this population, as well as an update on the latest evidence for the management of CMV infection.

## Introduction

Hematopoietic stem cell transplantation (HSCT) has significantly increased survival in patients suffering from hematological malignancies, hemoglobinopathies, and other hematological disorders [1]. Nonetheless, these patients are at high risk of complications, notably infections and rejection. Cytomegalovirus (CMV) is one of the most encountered infections in this population, with considerable implications on morbidity and mortality [2].

1. **Why is CMV relevant in HSCT?**
  HSCT patients experience a transient and prolonged immunocompromised state, during which their T-cell responses are suppressed, making them highly susceptible to viral infections such as CMV [3]. In fact, up to two-thirds of allogeneic HSCT (allo-HSCT) patients are at risk of developing CMV infection [4]. If left untreated, end-organ disease may develop in up to 30% of these patients [5], with high mortality rates reaching 37% within 6 months and close to 70% when CMV pneumonitis is present [3, 6]. Donor and recipient CMV seropositivity has been found to be an independent risk factor affecting leukemia-free survival and overall survival, even in the absence of end-organ disease [7]. In addition, CMV is believed to be an immunomodulatory virus [7–9]. Indeed, HSCT patients with CMV infection are more susceptible to life-threatening bacterial and fungal infections [10, 11]. Despite all the progress made in the prevention and treatment of CMV infection in HSCT recipients [12], mortality remains high. Moreover, refractory, resistant, and late CMV infection are all ongoing challenges that warrant attention [8, 13].
2. **Which HSCT recipients are at increased risk for CMV infection?**
  Donor and recipient serostatus are major risk factors for reactivation of the CMV virus from latency. Seropositive recipients of seronegative donors (D −/R +) are at the highest risk [14], followed by seronegative recipients of seropositive donors (D + /R −)[15], then (D + /R + ) recipients and (D −/R −) recipients [16]. Delayed immune reconstitution and impaired CMV-specific cellular immunity are also associated with increased risk of reactivation [17]. For instance, T-cell depleting agents such as alemtuzumab and anti-thymocyte globulin (ATG) which are usually given to HSCT recipients, have been found to increase the risk of CMV infection by 4.8- and 1.4-folds, respectively [18]. Other established risk factors include development of graft-versus-host disease (GVHD) necessitating augmentation of immunosuppressive regimens, such as high-dose corticosteroids and mycophenolate mofetil [13, 19], fludarabine and 2-chlorodeoxyadenosine [20–23], total body irradiation (TBI), and T-cell depleted grafts. Finally, despite reducing the risk of early CMV disease post-transplant, prophylactic and preemptive therapy have been shown to increase the risk of late CMV reactivation. This is believed to be caused by a reduction in the viral antigen load which delays the establishment of an efficient CMV-specific immune response [24, 25].
3. **Which HSCT recipients need prophylaxis for CMV infection?**
  CMV replication is associated with increased mortality independently of end-organ disease [26]. Thus, primary chemoprophylaxis is desirable to prevent CMV reactivation in high-risk individuals. Prior to initiating prophylaxis, clinicians should assess the risk of reactivation of CMV in the host and take into consideration potential drug toxicity and drug–drug interactions. Historically, the efficacy of prophylaxis using ganciclovir and foscarnet has been limited by serious adverse effects like myelosuppression and nephrotoxicity. However, the advent of newer and much safer agents is driving primary prophylaxis to become the standard of care [27]. Patients who would benefit from primary chemoprophylaxis include CMV seropositive recipients, seronegative recipients who receive a T cell-depleted graft from a seropositive donor, recipients of an HLA-mismatched or an umbilical cord blood allograft, and patients who receive alemtuzumab, ATG, or post-transplant cyclophosphamide [18, 28, 29].
4. **Which drugs can be used for CMV prophylaxis in HSCT?**
  A meta-analysis comparing six antiviral drugs used for CMV prophylaxis in HSCT showed that the most effective agents at reducing CMV reactivation and disease were ganciclovir and letermovir [30]. Ganciclovir is a synthetic nucleoside analogue that inhibits viral replication through the DNA polymerase and is highly active against herpesviruses [31]. Ganciclovir is given at a dose of 5 mg/kg twice daily for the first 5–7 days, followed by 5 mg/kg once daily [20]. However, despite decreasing rates of reactivation, it does not reduce mortality and is associated with a high incidence of neutropenia and high rates of secondary bacterial and fungal infections [32, 33]. On the other hand, letermovir is a relatively new drug approved by the FDA in 2017 for primary CMV prophylaxis in seropositive HSCT patients. Letermovir inhibits CMV replication by binding to components of the terminase complex (UL51, UL56, or both). Unlike ganciclovir, it is not active against other herpesviruses and should be co-administered with aciclovir or valaciclovir for prophylaxis against *Herpes simplex* and *Varicella zoster* virus reactivation [34]. In a phase 3 clinical trial, letermovir showed significant decrease of CMV infection at 24 weeks after HSCT, with no reported myelotoxicity, as compared with placebo, with a significant reduction of mortality to 5.7% at 24 weeks [24]. In addition, recently published real-world data also showed a higher 6-months overall survival rate in patients who received letermovir as primary CMV prophylaxis [35]. Moreover, letermovir prophylaxis has been associated with a significant decrease in the incidence of resistant and refractory CMV disease [36]. Finally, there are currently no data to support the use of maribavir for CMV prophylaxis as compared to placebo, as it did not show superiority at preventing CMV disease in the phase 3 clinical trial [37].
5. **What is CMV preemptive therapy and which HSCT recipients need it?**
  Pre-emptive therapy (PET) consists of surveillance of CMV reactivation with weekly polymerase chain reaction (PCR) tests and initiation of antiviral agents once viremia is detected at a pre-defined threshold. There is no consensus about the threshold PCR level to initiate PET, as studies suggest different cut-off points [5, 38]. In addition, whether the first episode of CMV DNAemia (called blip) reflects a risk of CMV disease, and whether it requires PET is still debatable [39, 40]. Clinicians should take the decision to initiate PET based on the patient’s profile and sero-status.Viral load is typically monitored for at least 100 days after transplant, or longer in patients with acute or chronic GVHD, or those with persistent T-cell immunodeficiency [41, 42]. The aim of this strategy is to prevent progression of CMV infection to end-organ-disease. Anti-CMV drugs are given for 2 weeks, and the duration may be extended for an additional week until CMV viral load is undetectable [42]. The first line for PET is usually ganciclovir, given at 5 m/kg intravenously (IV) every 12 h, or valganciclovir (prodrug of ganciclovir) at 900 mg orally twice daily, with close monitoring of the cell counts. Foscarnet at 60 mg/kg IV every 12 h and cidofovir 5 mg/kg IV once per week can be used as second and third-line agents, in particular when there is concern for drug-drug interactions or worsening bone marrow suppression [42]. However, both drugs are associated with significant toxicities, particularly nephrotoxicity [42].
6. **How to decide between PET and prophylaxis for CMV prevention in HSCT?**
  Studies have shown no difference in the risk of CMV disease between patients who received ganciclovir prophylaxis and PET [26, 43]. In addition, two systematic reviews demonstrated no reduction in all-cause mortality in patients who received ganciclovir and valganciclovir as prophylaxis [30]. Consequently, in view of (val)ganciclovir’s myelotoxicity, and foscarnet’s nephrotoxicity, PET was the recommended strategy for prevention of CMV disease [42].
  However, when letermovir was introduced, it was shown to significantly decrease CMV reactivation and improve patients’ survival in the first 24–48 weeks post-transplant, with a favorable safety profile, particularly without any myelotoxicity or nephrotoxicity [24]. Moreover, in a comparative study on the use of letermovir in CMV-seropositive recipients undergoing haplo-HSCT, it was shown to be more effective than PET [24]. Therefore, when the drug is available, it should be the preferred approach to prevent CMV disease in this high-risk population.
  The decision between PET and chemoprophylaxis in other HSCT recipients should consider individual patient’s risk factors for developing CMV infection, and the chemotherapeutic regimen. One should calculate the risks versus benefits of each strategy, taking into account drug-related toxicities, costs, and drug-drug interactions. Of note, when choosing the PET approach, compliance of the patient with routine monitoring of CMV viral load can sometimes be challenging.
7. **How to treat CMV infection in HSCT recipients?**
  The first line therapy for CMV viremia and end-organ disease including pneumonitis, colitis, and retinitis is ganciclovir at a dose of 5 mg/kg IV every 12 h. Valganciclovir is a prodrug of ganciclovir, with 60% bioavailability, and can be used at a dose of 900 mg every 12 h when there is no concern about gastrointestinal (GI) absorption (like in GI GVHD) [44]. Myelosuppression is a major side effect of (val) ganciclovir [45]. Foscarnet at a dose of 90 mg/kg IV every 12 h or 60 mg/kg IV every 8 h, or cidofovir 3–5 mg/kg per week for 2 weeks followed by 5 mg/kg every other week may be given as second and third lines of treatment, respectively, when bone marrow suppression worsens or in the setting of CMV viral resistance [42]. However, nephrotoxicity and electrolyte abnormalities are major side effects of foscarnet and cidofovir [46, 47]. The duration of treatment is usually 2–3 weeks. Most importantly, it should be individualized according to the host’s profile, clinical and virologic response [48]. Evidence regarding the use of CMV hyperimmune globulins (CMVIG) is limited to CMV pneumonitis, and should be considered whenever available, particularly in patients failing to respond to antiviral therapy [49]. As for CMV meningo-encephalitis, a combination of IV ganciclovir at a dose of 5 mg/kg every 12 h with foscarnet at a dose of 90 mg/kg daily is usually given; however, the evidence behind this strategy is mostly based on case series and experts' opinion.
8. **What is the difference between refractory CMV infection and resistant CMV in HSCT recipients?**
  Resistant CMV infection is defined by the presence of viral genetic mutations which decrease the susceptibility of the virus to anti-CMV drugs. Testing for CMV resistance is done with genotypic assays [50]. These are less likely to detect resistant viral strains when the CMV viral load is lower than 2000 IU/mL [51]. Moreover, resistance may differ according to the site of CMV disease [52]. On the other hand, refractory CMV infection refers to clinical resistance, which can be secondary to host or viral factors. There are specific proposed definitions for resistant and refractory CMV to be used in future clinical trials. Refractory/resistant infection should be suspected if there is an increase or persistence of CMV DNAemia after appropriate drug therapy, or if there is progression towards end-organ disease (GI, lungs, retina, or central nervous system) while on CMV treatment [53]. Refractory CMV disease is more common than resistant CMV and can occur anytime following HSCT, as opposed to resistant CMV, which is uncommon during the first 6 weeks of transplant, in particular if the patient has not been previously exposed to anti-CMV medications [36, 54]. It is very important to distinguish between these 2 entities, due to the differences in the management strategies, and since the mortality rate of resistant CMV may reach up to 42% [55].
  Risk factors for resistant/refractory CMV include previous exposure to anti-CMV drugs in the setting of a replicating virus, prolonged treatment for CMV disease, haploidentical and T-cell depleted HSCT [40].
9. **How to manage resistant/refractory CMV disease in the setting of HSCT?**
  Patients with resistant/refractory CMV infection should be managed by an expert infectious disease specialist. When suspected, immunosuppressive agents should be reduced whenever possible, and the anti-CMV drug should be switched awaiting genotypic testing [56]. Prior drug exposure and toxicity profile are additional factors that should be considered when selecting the appropriate antiviral regimen. Mutations in UL97, which encodes for the viral kinase, are frequently associated with ganciclovir resistance. Switching to foscarnet, if high-level UL97 resistance mutations are found (> fivefold increase in ganciclovir IC50), is recommended. However, certain low-level UL97 resistance mutations (M460I, C592G, L595W) can be managed with a higher-dose of ganciclovir at 7.5–10 mg/kg every 12 h [48]. Close monitoring and administration of granulocyte colony-stimulating factors may be used pre-emptively when such doses of ganciclovir are given [57].
  Mutations in the UL45 which encodes for CMV DNA polymerase indicate foscarnet resistance or cross-resistance to foscarnet, ganciclovir, and/or cidofovir. According to the level of resistance, switching to cidofovir or ganciclovir might be warranted. If cross-resistance to all three classes of drug is suspected, administering a combination of foscarnet and high dose ganciclovir (7.5–10 mg/kg every 12 h) is an option [56].
  Maribavir, a pUL97 kinase inhibitor, was recently approved in the United States for the treatment of post-transplant refractory/resistant CMV infection in patients who are older than 12 years of age and who weigh more than 35 kg [58]. In a phase 3 trial examining resistant/refractory CMV disease, maribavir was found to be superior to investigator-initiated therapy with studied outcomes of 8-weeks CMV clearance, sustained 16-weeks clearance, symptom control and tolerability [59]. It is worth mentioning that maribavir has limited penetration into the blood–brain barrier, which limits its use in CMV meningo-encephalitis.
  There are limited data supporting the use of letermovir in refractory/resistant CMV infection. Although letermovir prophylaxis has been associated with a reduced risk of refractory/resistant infection [42], many studies reported a mutation of the UL56 that is associated with a low threshold for the development of resistance [60–62]. However, letermovir may be useful in resistant/refractory disease in patients with a low viral load.
  The recommended treatment duration for resistant/refractory CMV infection is 2–4 weeks. The duration should be guided by clinical response and achievement of two consecutive undetectable CMV PCR in blood [56].
10. **Do cellular adoptive therapies play a role in the management of CMV in HSCT?**
  Significant in-vitro advancements have been made with T-cell therapy for the management of resistant/refractory CMV infection. Until now, this strategy is only recommended as an adjunct therapy, in view of the limited data from randomized clinical trials. It is an appealing approach, because adoptive cellular therapies may accelerate immune reconstitution.

There are several methods for generating virus-specific T cells, including stimulating virus-specific cells with a viral protein, followed by using these cells in vivo for further growth or directly infusing them into the recipient [63]. T-cells can be isolated from a CMV seropositive matched donor or from a third-party. Multiple infusions may be required, especially if the initial response is inadequate or if CMV viremia rebounds [64]. However, the process can take 4–6 weeks, with significant logistical challenges, and is therefore inconvenient for prompt management of resistant/refractory CMV disease [65].

A recent review of adoptive T-cell therapy for various dsDNA viruses in allo-HSCT recipients showed that 516 patients were reported to have used this strategy with 71% of them achieving partial or complete response [66]. Further randomized trials are needed to determine the efficacy and safety of adoptive immunotherapy for the management of CMV infection in HSCT recipients.

## Conclusion

Clinicians dealing with HSCT recipients should be mindful that CMV infection is associated with worsened outcomes, and should maintain a high index of suspicion and apply evidence-based strategies in the prevention and treatment of CMV infection and disease to improve patients’ survival.

A variety of approaches are currently available for the management of CMV infection, even in the setting of refractory/resistant disease. For a long time, PET was considered the standard of care, due to potential drug toxicity associated with antiviral agents used for prophylaxis. With the advent of newer agents with a favorable safety profile, and the accumulating evidence of their long-term benefits, prophylaxis may emerge once again as the new standard of care instead of PET. Adoptive T-cell therapy and hyperimmune globulins have a potential role in the treatment of challenging cases and need to be further investigated as an added treatment modality.

## Data Availability

Data sharing is not applicable to this article as no datasets were generated or analyzed during the current study.

## Abbreviations

ATG: Anti-thymocyte globulin
CMV: Cytomegalovirus
CMVIG: Cytomegalovirus hyperimmune globulins
D +: CMV seropositive donor
D-: CMV seronegative donor
FDA: Food and drug association
GI: Gastrointestinal
GVHD: Graft versus host disease
HLA: Human leukocyte antigen
HSCT: Hematopoietic stem cell transplantation
IC50: Half maximal inhibitory concentration
PCR: Polymerase chain reaction
PET: Preemptive therapy
R +: CMV seropositive recipient
R −: CMV seronegative recipient
allo-HSCT: Allogeneic hematopoietic stem cell transplantation
auto-HSCT: Autologous hematopoietic stem cell transplantation
dsDNA: Double stranded DNA
